# Exploiting semantic information in a spiking neural SLAM system

**DOI:** 10.3389/fnins.2023.1190515

**Published:** 2023-07-05

**Authors:** Nicole Sandra-Yaffa Dumont, P. Michael Furlong, Jeff Orchard, Chris Eliasmith

**Affiliations:** Centre for Theoretical Neuroscience, University of Waterloo, Waterloo, ON, Canada

**Keywords:** simultaneous localization and mapping, semantic SLAM, path integration, spiking neural networks, neuromorphic, hyperdimensional computing, neural engineering framework, semantic mapping

## Abstract

To navigate in new environments, an animal must be able to keep track of its position while simultaneously creating and updating an internal map of features in the environment, a problem formulated as simultaneous localization and mapping (SLAM) in the field of robotics. This requires integrating information from different domains, including self-motion cues, sensory, and semantic information. Several specialized neuron classes have been identified in the mammalian brain as being involved in solving SLAM. While biology has inspired a whole class of SLAM algorithms, the use of semantic information has not been explored in such work. We present a novel, biologically plausible SLAM model called SSP-SLAM—a spiking neural network designed using tools for large scale cognitive modeling. Our model uses a vector representation of continuous spatial maps, which can be encoded via spiking neural activity and bound with other features (continuous and discrete) to create compressed structures containing semantic information from multiple domains (e.g., spatial, temporal, visual, conceptual). We demonstrate that the dynamics of these representations can be implemented with a hybrid oscillatory-interference and continuous attractor network of head direction cells. The estimated self-position from this network is used to learn an associative memory between semantically encoded landmarks and their positions, i.e., an environment map, which is used for loop closure. Our experiments demonstrate that environment maps can be learned accurately and their use greatly improves self-position estimation. Furthermore, grid cells, place cells, and object vector cells are observed by this model. We also run our path integrator network on the NengoLoihi neuromorphic emulator to demonstrate feasibility for a full neuromorphic implementation for energy efficient SLAM.

## 1. Introduction

Simultaneous localization and mapping (SLAM) is the computational process of keeping track of one's location while navigating an unknown environment (i.e., *localization*) and, simultaneously, creating a map of the environment (i.e., *mapping*). Accurate localization is required for building metric map from egocentric observations, but errors in localization accumulate when relying solely on internally generated signals or self-motion (i.e., path integration or dead reckoning). An allocentric environment map can be used to correct these errors, making localization and mapping interdependent processes. SLAM is a core problem in mobile robotics, particularly in applications where high-precision GPS data is not available, such as in autonomous underwater vehicles or planetary exploration (Kim and Eustice, 2013; Palomeras et al., 2019; Geromichalos et al., 2020).

Biological systems have evolved to solve these problems. Animals are capable of navigating and creating maps of novel environments, deducing their current location, and retracing their steps. Considerable research has been conducted to investigate the neural mechanisms underlying spatial cognition in animals. It is known that many animals—including rodents (Mittelstaedt and Mittelstaedt, 1982; Etienne, 1987; Benhamou, 1997), bats (Aharon et al., 2017), and humans (Mittelstaedt and Mittelstaedt, 2001) – are capable of path integration. Tolman (1948) proposed that animals construct “cognitive maps”: internal mental constructs used to retain and retrieve information about the relative locations and features of an environment. Such maps are widely believed to be used to discover novel shortcuts and provide corrections to path integration, much like SLAM systems in robots. Indeed, animals have access to a plethora of external sensory information, such as visual landmarks and odor trails, which can be used to correct the errors that would accumulate when using path integration alone. The hippocampal formation is believed to be crucial for such computations, with place cells, head direction cells, and grid cells thought to play significant roles. In fact, Safron et al. (2022) have characterized the hippocampal-entorhinal system as “the most sophisticated of all biological SLAM mechanisms”.

While SLAM is a well-studied problem, and modern mobile robots are capable of performing SLAM, animal navigational abilities are still superior; they are more robust, efficient and adaptive, making them more useful in challenging real-world environments. Animals can use information from multiple sensory modalities (e.g., visual, olfactory, auditory, magnetoreception, and idiothetic cues) for navigation. Additionally, animals are able to navigate and map their environment in real-time using power-constrained computational resources, which is something that robots are still not able to achieve—brains are far more energy efficient than the GPUs or CPUs used to execute typical SLAM algorithms. The brain consumes around 20 Watts of energy while a single modern graphics card requires around 350 Watts. By taking inspiration from biology, researchers are trying to develop SLAM algorithms that are optimized for online processing, and that can run on resource-constrained platforms. For instance, neuromorphic hardware—designed to mimic the functionality of biological neural networks—is particularly well-suited for resource-constrained computing because it is designed to be energy-efficient and can perform brain-like computations using minimal resources (Bersuker et al., 2018; Thakur et al., 2018; Rathi et al., 2021).

Biology has influenced the development of a new category of SLAM models that includes RatSLAM (Milford et al., 2004), DolphinSLAM (Silveira et al., 2015), and NeuroSLAM (Yu et al., 2019), among others. Remarkably, some of these models have demonstrated performance comparable to contemporary state-of-the-art approaches. However, this is still an active area of inquiry, with questions remaining regarding scalability and biological plausibility of these approaches, as well as their deployability on neuromorphic hardware. While these types of SLAM algorithms have made notable progress, they have yet to fully explore the wealth of knowledge available from neuroscience and cognitive science. Animals extract and make use of higher-order semantic information about their environment and landmarks from raw sensory inputs while navigating. Recent advancements in robotics have successfully incorporated semantic information into SLAM models (Bowman et al., 2017; Zhang et al., 2018; Chen et al., 2019; Fan et al., 2022). Semantic SLAM models use deep neural networks to extract semantic information to build environment maps. By utilizing higher-level conceptualization of states grounded in cognitive meaning, these models can augment and improve upon purely metric SLAM. Consequently, the construction of maps containing semantic representations empowers such SLAM systems to interact with environments in sophisticated and intelligent ways.

In the same way that biology can aid in the development of AI and robotics, computational modeling can also provide valuable insights into biological research questions. By creating computational models of SLAM that are constrained to be biologically plausible, we can gain a deeper understanding of the neural algorithms that may underlie spatial cognition in animals. For example, we can investigate hypotheses on how exactly cognitive maps may be learned, stored, and used to assist in navigation. Or how multi-modal sensory information is integrated during the construction of cognitive maps. Or how such maps may be accessed and queried to reason about space.

In this work, we unite biologically inspired and semantic SLAM in our model SSP-SLAM, and consider how our computational model can explain neuroscientific observations. Specifically, we present a novel spiking neural network SLAM system, called SSP-SLAM. This model is built using the Neural Engineering Framework (NEF) (Eliasmith and Anderson, 2003) and the Semantic Pointer Architecture (SPA) (Eliasmith, 2013). The NEF provides a systematic method for embedding a state space model into a spiking neural network that can run on neuromorphic hardware. The SPA, which includes Spatial Semantic Pointers (SSPs), provides an approach for representing and processing symbol-like information in connectionist systems. The SPA provides an architecture and “semantic pointer” representations, for characterizing neural processing, including that of symbols, as manipulation of high-dimensional vectors. This enables the development of systems that can learn and reason about symbolic information in a scalable, differentiable, and compositional manner. These methods are used in SSP-SLAM to build environment maps. These maps are core to the functioning of SSP-SLAM, as they integrate semantic information, while being combined with an SSP-based path integrator. The resulting model provides the following contributions:

- We propose and implement a novel spiking neural network SLAM model.
- We constrain our model to only use quantities that are known to be represented in hippocampus, like spatial representations of head direction cells, object vector cells, place cells, and grid cells. Furthermore, biologically plausible, Hebbian-like rules are used to learn an environment map in the form of an associative memory.
- We explore compositional semantic map representations using the SPA and the principles of vector symbolic architectures more broadly. We demonstrate how such a map can be queried post-training to recall what landmarks were in particular areas, recall where landmarks of certain types or colors were located, and compute (online) the vector between self-position and landmarks in memory.
- We illustrate first steps toward a neuromorphic implementation of our model, showing that the path integration component of SSP-SLAM can be run on an emulator of Intel's Loihi neuromorphic chip.

## 2. Materials and methods

### 2.1. The semantic pointer architecture

Our computational SLAM model is built using the tools and principles of the semantic pointer architecture (SPA). This framework has been used to model various cognitive processes, such as action selection (Stewart et al., 2012b), planning (Blouw et al., 2016), memory and free recall (Gosmann and Eliasmith, 2021), and reinforcement learning (Rasmussen and Eliasmith, 2014; Duggins et al., 2022). Furthermore, it has been used to construct a large-scale functional brain model, Spaun (Eliasmith et al., 2012; Choo, 2018), with over 6 million neurons and 20 billion connections. The SPA proposes that *semantic pointers* are the fundamental representations of biological cognition (Eliasmith, 2013). Semantic pointers are spiking neural implementations of high-dimensional vectors that are defined by their compression relations to other neural representations. In the case of cognitive semantic pointers, they can be used to represent concepts, objects, or states, and can be combined in a distributed and compositional manner to represent more complex meanings or structures. By means of the neural engineering framework (NEF), semantic pointers in the SPA are generated by the activities of a collection of spiking neurons. Operations on the underlying vectors are then performed through setting the connections within the spiking neural network. As such, the SPA provides a means to translate symbolic cognitive models into biologically plausible spiking neural networks, and is an approach to neurosymbolic AI. As we will demonstrate, it can be used to build a spiking neural network SLAM model that is deployable on neuromorphic hardware.

#### 2.1.1. Algebra of cognition

The cognitive representations in the SPA are based on *hyperdimensional computing*, also known as *vector symbolic architectures* (VSAs), which bridge symbolic and connectionist approaches to AI. A VSA is any computing framework in which symbols and structured compositions of symbols are represented as high-dimensional vectors. The VSA includes a set of algebraic operations, defined over the vector space, that correspond to operations on the underlying symbols, effectively creating an algebraic language for cognition. The key operations that define this algebra are a similarity measure, a hiding operation, a bundling operation, a binding operation, and an inverse operation. The specification of these operations differentiates particular VSAs. In this work, we implement the SPA using Holographic Reduced Representations (HHRs; Plate, 1995), realized in spiking neural networks.

The *similarity measure* between two semantic pointers indicates the semantic similarity of the symbols they represent. This is given by the cosine similarity (or dot product), which is also the measure for semantic similarity used in many vector encodings in machine learning (Mikolov et al., 2013). The *bundling* operation is addition, and is used to group semantic pointers in a set. The *binding* and *hiding* operations are used to combine symbols together (e.g., combining a slot and filler, to have a single slot-filler representation). In HRRs, binding and hiding are done by one operation, circular convolution,


(1) $$\begin{array}{l} A⊛B=F^{-1}\left\{F\left\{A\right\}⊙F\left\{B\right\}\right\}, \end{array}$$

where F is the Discrete Fourier Transform (DFT), and ⊙ is the Hadamard product. The *inverse* operation takes a single input vector and produces a single output vector that reverses the effect of binding with the input vector, (*A*⊛*B*)⊛*B*^−1^ = *A*. In HRRs, an easy-to-compute and numerically stable approximate inverse (involution) is frequently used. It is defined as $B^{-1}=\left[B_{1},B_{d},B_{d-1},\ldots ,B_{2}\right]$.

To understand how these operations are used to compose and reason about structured representations, consider a concrete example. Let *X* denote the semantic pointer representation of the concept X. The sentence, “a brown cow jumped over the moon”, can thus be represented via binding and bundling operations as follows:


(2) $$\begin{array}{c} SUBJECT⊛(COLOR⊛BROWN+ANIMAL\\ ⊛COW)+VERB⊛JUMP+OBJECT⊛MOON \end{array}$$

The semantic pointer representations of various slots (e.g., subject, color, verb) are bound with the semantic pointers representations of various fillers (e.g., cow, jump), all of which are summed together to represent their collection in a single sentence. The final vector can be queried via the inverse operation to retrieve information. For example, by binding the final vector with *VERB*^−1^ we can approximately obtain the semantic pointer *JUMP*.

Typically, VSAs have been used to represent discrete symbols with a one-to-one mapping used to translate between symbols and vectors. Random high-dimensional vectors are often used. Certain models have employed machine learning techniques to obtain vector embeddings with desired similarity characteristics (Mitrokhin et al., 2020). In recent years, VSAs have been extended to represent continuous features using a mapping conceived as a fractional version of the binding operator.

#### 2.1.2. Spatial semantic pointers

Spatial Semantic Pointers (SSPs) extend VSAs to support representation of continuous features (Komer et al., 2019). Here, the mapping from input features to an output vector, ℝ^*m*^ → ℝ^*d*^, is explicitly defined. A *d*-dimensional SSP representing an *m*-dimensional variable **x** is given by


(3) $$\begin{array}{lll} \phi \left(\text{x}\right) & = & F^{-1}\left\{e^{iA\text{x}}\right\} \end{array}$$

where *A*∈ℝ^*d*×*m*^ is the encoding matrix of the representation, *A*x is a *d*-vector, and *e*^*iA*x^ is a vector of *d* complex numbers. The dot products of x with a fixed set of *d* vectors—the rows of the encoding matrix—are cast as the phases of complex exponentials to obtain the high dimensional SSP useful for hyperdimensional computing. There is freedom in the selection of this matrix. However, to ensure the SSP is real-valued, the encoding matrix must be chosen so that *e*^*iA*x^ is conjugate symmetric. Though originally SSPs were developed as a fractional extension to the binding operator of HRRs, the resulting mapping is similar to the encoding used in Random Fourier Features (RFF), a popular method for approximating kernels in machine learning (Rahimi and Recht, 2007; Furlong and Eliasmith, 2022).

A useful property of SSPs is that binding in the SSP space is equivalent to addition in the variable space,


(4) $$\begin{array}{l} \phi \left(\text{x}\right)⊛\phi \left({\text{x}}^{\prime }\right)=F^{-1}\left\{e^{iA\text{x}}⊙e^{iA{\text{x}}^{\prime }}\right\}=\phi \left(\text{x}+{\text{x}}^{\prime }\right). \end{array}$$

Thus, it is easy to “update” SSP representations without any decoding. For instance, it is easy to “move” an object located somewhere with one or more binding operations.

**Figure 1:**
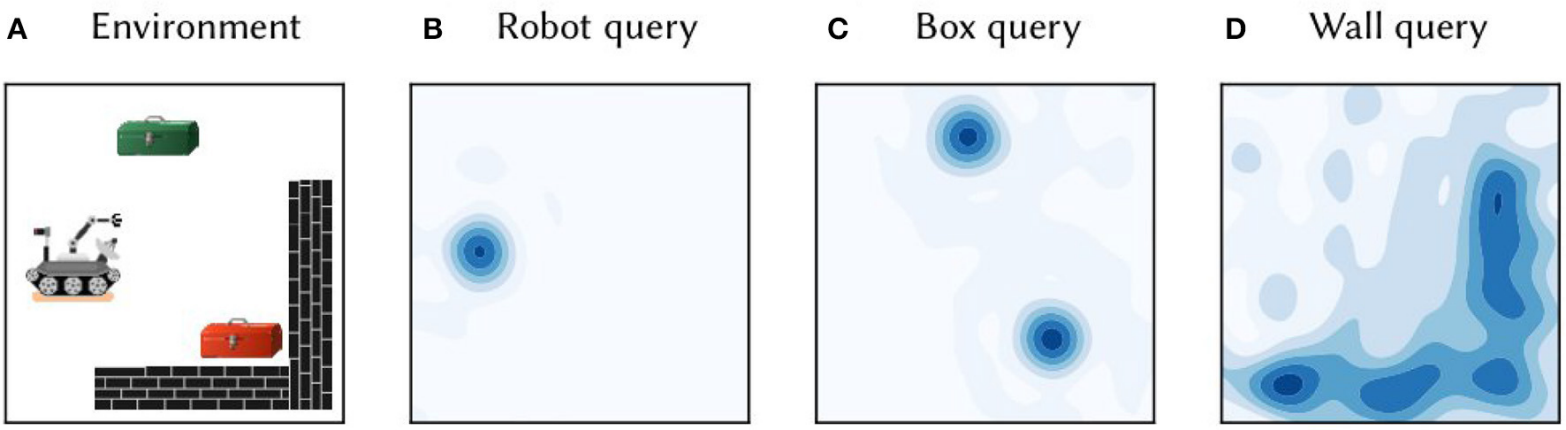
**(A)** A toy 2D environment consisting of a robot, boxes, and walls. Information about the objects and their locations was encoded in single vector *M*, as per Equation (5). **(B)** The vector *M* was queried for the location of the robot by approximate unbinding: $M⊛{ROBOT}^{-1}\approx \phi \left(x_{1},\;y_{1}\right)$. The heat map shows the cosine similarity between the query output and SSP representations of points gridded over 2D space. **(C)** The similarity map obtained from querying the map *M* for the location of boxes. **(D)** The similarity map obtained from querying for the wall area.

Generally, the SSP representation of a number is similar to nearby numbers (in terms of Euclidean distance), and dissimilar to distant ones—with some rippling effects. As a result, similarity between SSPs provides a method for visualizing these high-dimensional vectors (see Figure 1). The similarities between a particular SSP and a set of SSPs that represent points gridded over *m*-dimensional space can be computed and plotted. We refer to such plots as similarity maps. For example, a similarity map of an SSP, ϕ′, representing a 1−D variable is a plot of *x* vs. ϕ′·ϕ(*x*), which has been shown to be a sinc function in the limit *d* → ∞ (Voelker, 2020). For SSPs representing 2D variables, a similarity map can be depicted as a surface plot or a heat map as in Figure 1.

A primary advantage of the SSP representation is that it can be used in combination with semantic pointers encoding discrete symbols. Figure 1 provides a concrete demonstration of such a representation. Consider a simple 2D environment consisting of different objects and landmarks: a robot, two boxes, and a wall (see Figure 1A). The position of the robot, (*x*_1_, *y*_1_), can be encoded as a SSP, ϕ(*x*_1_, *y*_1_). This, in turn, can be bound (i.e., circularly convolved) with the semantic pointer representing the *concept* of a robot, *ROBOT*, to obtain *ROBOT*⊛ϕ(*x*_1_, *y*_1_)—this represents a robot at a particular location. Likewise, the semantic pointer for a tool box can be bound with SSPs encoding their locations, (*x*_2_, *y*_2_) and (*x*_3_, *y*_3_), to obtain *BOX*⊛(ϕ(*x*_2_, *y*_2_)+ϕ(*x*_3_, *y*_3_)); in this case, the sum of SSPs is used to represent a set of locations. The wall in the environment covers an area *D*, which can be represented by integrating the SSP encoding over that area, ∬_*D*_ϕ(*x, y*)*dxdy*. All together, the complete environment can be represented by adding all of these object-location vector encodings:


(5) $$\begin{array}{r} \begin{array}{r} M=ROBOT⊛\phi (x_{1},\;y_{1})+BOX⊛\left(\phi (x_{2},\;y_{2})+\phi (x_{3},\;y_{3})\right) \end{array}\\ \begin{array}{l} +WALL⊛\iint_{D}\phi (x,\;y)dxdy \end{array} \end{array}$$

This vector was constructed and “queried” for different locations with approximate unbinding. The results of this unbinding are shown in Figures 1B–D. The high-dimensional SSPs are visualized in this figure via their similarity to neighboring points.

#### 2.1.3. Probability representations

Recent work has demonstrated that the algebra of cognition defined by VSAs and SSPs has a probabilistic interpretation (Furlong and Eliasmith, 2022). Using the tools provided by the NEF, it is possible to construct spiking neural networks that embody probability distributions and perform computations related to probability, such as determining entropy and mutual information (Furlong et al., 2021; Furlong and Eliasmith, 2023).

In particular, SSPs can be used for kernel density estimation (KDE), a non-parametric method used for estimating a probability density function of a random variable *X*. To approximate a PDF *f* given a set of samples, {x_1_, x_2_, …, x_*n*_}, drawn from an unknown distribution, one can average kernel functions around each data point, *k*(x − x_*i*_), to obtain a smooth estimate, $\hat{f}$, of the underlying PDF:


(6) $$\begin{array}{l} \hat{f}\left(\text{x}\right)=\frac{1}{n}\overset{n}{\underset{i=1}{\sum }}k\left(\text{x}-{\text{x}}_{i}\right). \end{array}$$

KDE has the advantage of being non-parametric and flexible, allowing the estimation of complex and multi-modal distributions. However, the choice of the kernel function is crucial for the accuracy and smoothness of the estimate. Common kernel functions used in KDE include the Gaussian, Epanechnikov, and triangle kernels.

The similarity, or dot product, between SSPs approximates a sinc kernel function. Consequently, we can define *k*(x − x_*i*_) = ϕ(x)·ϕ(x_*i*_). Our KDE is given by $\frac{1}{n}\overset{n}{\underset{i=1}{\sum }}\phi \left(\text{x}\right)\cdot \phi \left({\text{x}}_{i}\right)=\phi \left(\text{x}\right)\cdot M_{n}$, where $M_{n}=\frac{1}{n}\overset{n}{\underset{i=1}{\sum }}\phi \left({\text{x}}_{i}\right)$ is the average over datapoint SSP representations. Unlike the kernels listed above, the normalized sinc can take on negative values, but it can be used to obtain probability densities with a simple correction,


(7) $$\begin{array}{l} \hat{f}\left(\text{x}\right)\approx {\left(\phi \left(\text{x}\right)\cdot M_{n}-\xi \right)}^{+} \end{array}$$

where ξ is a fixed scalar chosen so that $\overset{\infty }{\underset{-\infty }{\int }}{\left(\phi \left(\text{x}\right)\cdot M_{n}-\xi \right)}^{+}d\text{x}=1$ (Glad et al., 2003, 2007). Note that this is simply a ReLU neuron with bias ξ, and either weights *M*_*n*_ and input ϕ(x), or vice versa—weights ϕ(x) and input *M*_*n*_. In the former case, a population of many such neurons (with varying incoming synaptic weights *M*_*n*_) can be interpreted as estimating the probability of a query ϕ(x) under different distributions. In the later case, the activities of a population of neurons would represent the probabilities of different sample points x under a given input distribution represented by SSPs, *M*_*n*_. Notably, the sinc estimate is often more accurate than the “optimal” Epanechinikov estimate (Section 1.3, Tsybakov, 2009).

Using SSPs for neural probability computations in this way results in different interpretations of the standard VSA operations, which are useful in the context of SLAM. Under this interpretation, bundling is used to add new datapoints to a running mean *M*_*n*_, and is a kind of belief update, binding can be used for multivariate KDE, and the inverse operation that performs unbinding is analogous to conditioning.

#### 2.1.4. The neural engineering framework

The SPA is not just a VSA, but rather a full architecture that includes a variety of functional components, as well as the neural instantiation of a VSA. To create spiking neural networks that implement algorithms involving VSAs and SSPs, we require methods to embed vector representations into the activity of spiking neurons, and to be able to perform computations on these vectors via projections between neural populations. The NEF provides such methods, which are described by three primary principles.

The first principle of *representation* specifies how the collective neural activity of a population encodes a vector and vectors can be decoded out of spike trains. The activity of neuron *i* in a population encoding a vector, ϕ∈ℝ^*d*^, is given by,


(8) $$\begin{array}{l} a_{i}\left(t\right)=G_{i}\left[\alpha_{i}e_{i}\cdot \phi +\beta_{i}\right], \end{array}$$

where α_*i*_>0 is the neuron's gain, β_*i*_ is its bias, ***e***_*i*_ is its encoder, and $G_{i}$ is a non-linear function—in this work, the leaky-integrate-and-fire (LIF) function. The gain and bias parameters vary amongst neurons to create a heterogeneous population. Encoders determine the type of input a specific neuron is responsive to, thus capturing the neuron's “receptive field”. In the case of a neural population representing SSPs, it is reasonable to set encoders as SSPs that represent random points in space. This produces a population of neurons that are sensitive to specific spatial locations—i.e., place cells. Other types of spatial sensitive neurons can be constructed using different neural encoders and SSP encoding matrices. In Dumont and Eliasmith (2020), grid cells were obtained this way.

A vector represented by the activity of a population of *N* neurons can be decoded from a linear combination of the spiking neural activity after post-synaptic filtering:


(9) $$\begin{array}{l} \hat{\phi }=\overset{N}{\underset{i=1}{\sum }}a_{i}\left(t\right)*h\left(t\right)d_{i}, \end{array}$$

where * is convolution and $d_{i}\in {\mathbb{R}}^{d}$ are called the decoders of the population. Least-squares optimization is typically used to solve for the decoders. The function *h*(*t*) is a post-synaptic filter and is parameterized by τ_*syn*_, the post-synaptic time constant:


(10) $$h(t)=\{\begin{array}{ll} e^{-t/\tau_{syn}} & \text{if }t>0\\ 0 & \text{otherwise}. \end{array}$$

The second principle of the NEF, *transformation*, provides the method for setting weights between two neural populations to compute a desired function. Assume a population of *N* neurons representing a vector, ϕ, is fully connected to a different population of *N*′ neurons. Suppose we would like second population to represent some function of the vector, *f*(ϕ). This function can be decoded out of the first population's activity,


(11) $$\begin{array}{l} \hat{f}\left(\phi \right)=\overset{n}{\underset{i=1}{\sum }}a_{i}\left(t\right)*h\left(t\right)d_{i}^{\left(f\right)}. \end{array}$$

These function-specified decoders, $d_{i}^{\left(f\right)}$, can be solved for using samples of the desired function output or, if sample outputs are not available, decoders can be learned online in response to error signals (see Section 2.1.5). Decoding the output of the first population and encoding it into the activity of the second population is equivalent to multiplying the filtered activities of the first population with a weight matrix and feeding that current into the second population, which will have activities given by


(12) $$\begin{array}{l} b_{j}\left(t\right)=G_{i}\left[\overset{N}{\underset{i=1}{\sum }}w_{ij}a_{i}\left(t\right)+\beta_{j}\right],\;w_{ij}=\alpha_{j}e_{j}\times d_{i}^{\left(f\right)} \end{array}$$

where × is an outer product.

The result is a standard neural network, with populations connected via weighted synapses. The NEF provides a method to generate the weight matrices that are the outer product between the decoders of the first population (which are optimized) and the encoders of the second (which are pre-set, usually to match biological tuning curves).

The last principle of the NEF is *dynamics*. Dynamical systems can be embedded into in a recurrently connected population of spiking neurons using this principle. The NEF proposes that to implement a non-linear dynamical system $\overset{•}{\phi }=f\left(\phi \right)+g\left(u\right)$ (where *u* is some input signal), the incoming connection from the population representing the input *u* must compute the transform τ*g*(*u*) (where τ is the post-synaptic time constant), and the recurrent connection from the population representing *S* to itself must compute the transform τ*f*(ϕ)+ϕ. This is due to the use of post-synaptic filters. This principle allows us to embed a wide variety of non-linear dynamical systems into spiking neural networks, which we exploit in Section 2.2.1.

#### 2.1.5. Learning rules

Biologically plausible learning rules that only use local information can be used in the NEF for modifying synaptic weights online. The Prescribed Error Sensitivity (PES) (MacNeil and Eliasmith, 2011) is an error-driven learning rule in which, to learn a connection between a pre- and post-population of neurons, the pre-population's decoders are modified in response to an error signal:


(13) $$\begin{array}{l} \Delta d_{i}=\kappa Ea_{i}, \end{array}$$

which is equivalent to modifying weights by


(14) $$\begin{array}{l} \Delta w_{ij}=-\kappa \alpha_{j}e_{j}\cdot Ea_{i} \end{array}$$

where κ is a learning rate, *a*_*i*_ are pre-population neural activities (filtered spikes), α_*j*_ are post-population gains, ***e***_*j*_ are the post-population encoders, and ***E*** is an error signal we seek to minimize. This signal may be computed by other neural populations in a model. Biologically, we can think of those populations as dopaminic neurons that can modify weights in this way via dopamine levels. Real data of spike timing dependent plasticity is matched by PES when used in combination with the unsupervised Bienenstock, Cooper, Munro (BCM) learning rule, which sparsifies weights (Bekolay et al., 2013).

Another, unsupervised, learning rule is the Oja learning rule (Oja, 1982), which modifies the Hebbian learning rule in order to improve stability. The vector version of this rule, the “Voja” learning rule, shifts encoders so that neurons fire selectively at particular inputs and activity is sparsified:


(15) $$\begin{array}{l} \Delta e_{i}=\kappa a_{i}\left(\text{x}-e_{i}\right). \end{array}$$

This rule has been used for training heteroassociative memory networks (Voelker et al., 2014), and is used in SSP-SLAM, along with the PES rule, to train an associative memory.

### 2.2. The SSP-SLAM model

In this paper, we develop a spiking neural network SLAM model using semantic pointers, SSPs and the NEF. The model, SSP-SLAM, consists of six main neural populations, grouped into four modules, that provide all the necessary functionality.

- **Localization module**

- *Path integrator:* A network maintaining an allocentric self-position estimate, represented as a SSP $\hat{\phi }\left(\text{x}\left(t\right)\right)$, that is dynamically updated using a velocity signal. Specifically, this is a recurrent neural network, consisting of many sub-populations representing controlled oscillators that contain heading direction cells.
- *Grid cell (GC) population:* A population representing a “cleaned-up" version of the SSP self-position estimate, $\phi \left(\hat{\text{x}}\left(t\right)\right)$.

- **Landmark perception module**

- *Object vector cell (OVC) population:* A population that encodes the SSP representation of distances and directions to landmarks and environmental features in view—i.e., an egocentric representation of feature locations.
- *Object location (OL) population:* A population that performs circular convolution to obtain an allocentric SSP representation of feature locations.

- **Environment map module**

- *Associative memory (AN) network:* A network that learns a mapping between landmarks and locations using the biologically plausible PES and Voja learning rules.

- **Loop closure module**

- *Map estimate (ME) population:* A population that performs circular convolution to obtain an alternative estimation of self-position using the environment map. This provides corrections to the path integrator.

**Figure 2:**
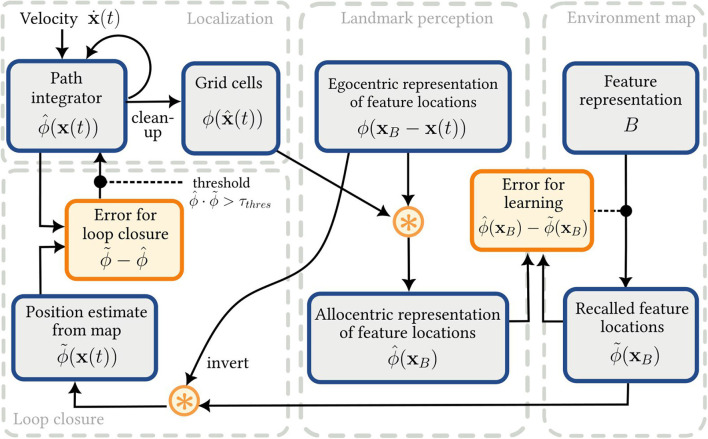
The SSP-SLAM model. Output of the localization module is used (along with the egocentric feature locations encoded by the OVC population) to train an associative memory network, which can be thought of as an environment map. The output of this map is, in turn, used for error correction of the PI model.

Each element of the SSP-SLAM model is described in more detail below and a high-level overview of the model is given in Figure 2.

#### 2.2.1. Localization module

In prior work, we have used SSPs to maintain a neural estimate of an agent's self-position while navigating an environment (Voelker et al., 2021; Dumont et al., 2022). To build a network that maintains an encoding of position, consider how ϕ(x) changes if x is a function of time. We can relate the rate of change of ϕ to the velocity $\overset{•}{\text{x}}\left(t\right)$:


(16) $$\begin{array}{ll} \overset{•}{\phi }\left(\text{x}\left(t\right)\right) & =F^{-1}\left\{e^{iA\text{x}\left(t\right)}⊙iA\overset{•}{\text{x}}\left(t\right)\right\}, \end{array}$$

where ⊙ is element-wise multiplication. Now consider the dynamics of an SSP in the Fourier domain. Taking the Fourier transform of Equation (16), we get,


(17) $$\begin{array}{ll} F\left\{\overset{•}{\phi }\left(\text{x}\left(t\right)\right)\right\} & =\left(iA\overset{•}{x}\right)⊙F\left\{\phi \left(\text{x}\left(t\right)\right)\right\}. \end{array}$$

Note that the dynamics of the Fourier components of an SSP are independent of one another. The dynamics of the *j*^th^ Fourier coefficient of the SSP can be written as


(18) $$\begin{array}{r} \begin{array}{r} \frac{d}{dt}[\begin{array}{c} \text{Re}ℱ{\left\{\phi (\text{x})\right\}}_{j}\\ \text{Im}ℱ{\left\{\phi (\text{x})\right\}}_{j} \end{array}]=[\begin{array}{cc} 0 & -\omega_{j}\\ \omega_{j} & 0 \end{array}][\begin{array}{c} \text{Re}ℱ{\left\{\phi (\text{x})\right\}}_{j}\\ \text{Im}ℱ{\left\{\phi (\text{x})\right\}}_{j} \end{array}], \end{array}\\ \begin{array}{l} \text{where }\omega_{j}\equiv A_{j,:}\cdot \overset{•}{\text{x}}(t) \end{array} \end{array}$$

Each Fourier coefficient of the SSP is thus a simple harmonic oscillator. The real and imaginary components of the Fourier coefficients of the SSP oscillate about the unit circle with time-varying frequency $\omega_{j}=A_{j,:}\cdot \overset{•}{\text{x}}\left(t\right)$. The oscillator frequencies are modulated by the velocity; in other words, they are velocity controlled oscillators (VCOs). In our model, we modify the dynamics of Equation (18) so that the unit circle is an attractor and the oscillators self-stabilize:


(19) $$\begin{array}{r} \begin{array}{r} \frac{d}{dt}[\begin{array}{c} \text{Re}ℱ{\left\{\phi (\text{x})\right\}}_{j}\\ \text{Im}ℱ{\left\{\phi (\text{x})\right\}}_{j} \end{array}]=[\begin{array}{c} -\omega_{j}\text{Im}ℱ{\left\{\phi (\text{x}(t))\right\}}_{j}+\frac{1-r_{j}^{2}}{r_{j}}\text{Re}ℱ{\left\{\phi (\text{x})\right\}}_{j}\\ \omega_{j}\text{Re}ℱ{\left\{\phi (\text{x}(t))\right\}}_{j}+\frac{1-r_{j}^{2}}{r_{j}}\text{Im}ℱ{\left\{\phi (\text{x})\right\}}_{j} \end{array}], \end{array}\\ \begin{array}{l} \text{where }r_{j}\equiv |ℱ{\left\{\phi \right\}}_{j}|. \end{array} \end{array}$$

This reduces drift and ensures the entire SSP vector remains unit length. Thus, our path integrator is a hybrid between continuous attractor and oscillatory inference models of path integration.

To realize this representation and dynamics in a spiking neural network, we use the tools of the NEF as described above. The SSP estimate of self-position is encoded in ⌊$\frac{d}{2}$⌋ recurrent populations of spiking neurons, each of which is a VCO. Only ⌊$\frac{d}{2}$⌋ VCO populations are needed since the Fourier transform of the SSP has conjugate symmetry (half of its Fourier components can be computed from the other half).

To compute the non-linearities bet ween the frequency and SSP Fourier coefficients, we must represent both in a single population, as is standard in the NEF. The vector being represented by the collective activity of the *j*^th^ VCO population is,


(20) $$\begin{array}{l} {\left[\begin{array}{ccc} \omega_{j} & \text{Re}F{\left\{\hat{\phi }\left(\text{x}\right)\right\}}_{j} & \text{Im}F{\left\{\hat{\phi }\left(\text{x}\right)\right\}}_{j} \end{array}\right]}^{T}. \end{array}$$

We write $\hat{\phi }\left(\text{x}\right)$ here to emphasize that this is an *estimate* of ϕ(x). Due to noise inherent in neural encoding and the dynamics being approximated by recurrent connections rather than being computed exactly, this estimate will drift from the SSP encoding of the ground truth position over time. Indeed, the vector encoded by the path integrator will even drift from the sub-space of SSP vectors in ℝ^*d*^ without some form of correction.

A population of speed- and heading-direction cells that encode the agent's velocity projects onto the VCO populations. The connection weights compute the linear transform needed to obtain the input frequencies, $A\overset{•}{\text{x}}\left(t\right)=\omega$. Each VCO neural population is recurrently connected to itself with weights optimized by least squares to implement the dynamics of Equation (19).

An advantage of this model is its ability to perform localization in space of different dimensionality without major modification. Consider the SSP representation of a x(*t*)∈ℝ^*m*^, given by ϕ(x(*t*))∈ℝ^*d*^, compared to an SSP of the same dimension *d*, but encoding a higher dimension variable, ϕ(***y***(*t*)) where ***y***(*t*)∈ℝ^*p*^ and *p*≠*m*. In either case, the dynamics of the SSP are given by Equation (18). The same set of VCO populations can model the dynamics of ϕ(x(*t*)) and ϕ(***y***(*t*)) – the only difference between their computations is the calculation of the frequencies, ω_*j*_, used in the VCOs. These frequencies are input to the path integrator, with incoming synaptic weights performing the linear transformation from self-motion to frequencies, $A\overset{•}{\text{x}}\left(t\right)$. The same path integrator network can receive input from multiple sources, with synaptic gating used to switch between localization in different dimensional spaces and coordinate frames.

Note that the VCO populations consist of spatially sensitive neurons, but these neurons will not resemble place or grid cells. Each oscillator is a population representing a frequency (derived from velocity) and a single Fourier coefficient of the SSP. This results in neurons with conjunctive sensitivity to heading direction, speed, and spatial position (in a periodic fashion, resembling a plane wave). Their firing patterns are velocity dependent bands or stripes. Banded cells have been predicted by other VCO models (Burgess, 2008) and have been a point of contention since reports of band cells in the hippocampal formation are limited, and their existence is controversial (Krupic et al., 2012; Navratilova et al., 2016). Additionally, grid cells do not intrinsically emerge from PI in the model presented in this section. Nevertheless, SSPs naturally represent grid cells, and we use such a population to represent the collective output of all VCOs after a clean-up operation and provide a better basis for the downstream construction of place cells and spatial maps (Orchard et al., 2013; Dumont and Eliasmith, 2020). This is not unwarranted, given the observations from the MEC. The deeper layers of the MEC receive hippocampal output [along with input from many other cortical areas (Czajkowski et al., 2013)], and is where head-direction cells, speed cells, and conjunctive grid cells are primarily located (Witter and Moser, 2006). The superficial layers of the MEC, specifically layer II, mainly provide input to the hippocampus and consist mostly of “pure” grid cells (Sargolini et al., 2006). This suggests that the deeper layers and head direction cells may play a crucial role in integrating external input, much like the path integrator network in SSP-SLAM. The output of this integration is then processed into more stable, purely spatial representations in the superficial layers, like the grid cell population in SSP-SLAM, which are used for downstream tasks. However, this narrative is subject to debate, and not universally accepted.

**Figure 3:**
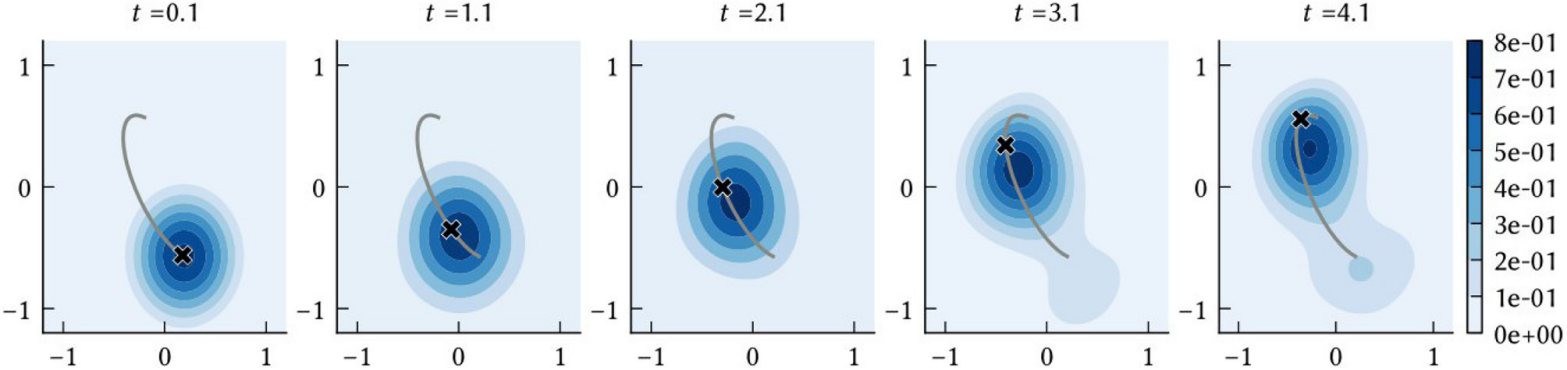
The time evolution of the posterior distribution *f*(x) = (ϕ(x)·ϕ(*t*)−ξ)^+^, where ϕ(*t*) is the output of the vector encoded by the collective activities of neurons in the path integrator network. The black “x” shows the ground truth x(*t*) at the sampled times, and the grey line shows the complete ground truth path.

As described in Section 2.1.3, SSPs can be used to construct probability distributions. When performing path integration, we are interested in obtaining an estimate of the agent's position at a given point in time. Let $\hat{\phi }\left(\text{x}\left(t\right)\right)$ be the vector represented by the path integrator network at time *t*. The network is initialized to encode the SSP ϕ(x(0)), from which a prior probability distribution can be computed. At every simulation time step this belief state is updated according to the dynamics given in Equation (19). Then, the probability density of the agent being at a location $\hat{\text{x}}$ is $\hat{f}\left(\hat{\text{x}}\right)\approx {\left(\phi \left(\hat{\text{x}}\right)\cdot \hat{\phi }\left(\text{x}\left(t\right)\right)-\xi \right)}^{+}$. The position estimate of the path integration model is taken to be the $\hat{\text{x}}$ that maximizes this posterior distribution, i.e., the maximum a posteriori probability (MAP) estimate. A simple example path decoded in this manner is shown in Figure 3. The SSP representation of the MAP estimate, $\phi \left(\hat{\text{x}}\right)$, is computed as a part of the “clean-up” process applied to the output of the VCOs to obtain the input to the grid cell population.

#### 2.2.2. Landmark perception module

In the SSP-SLAM model, the agent not only receives a self-velocity signal as input, but additionally receives observations of its local environment. As an animal moves through space, sensory systems and other brain regions provide information about its surroundings. The inferotemporal cortex, for example, plays a vital role in object recognition (Rajalingham and DiCarlo, 2019), and populations in the medial entorhinal cortex (MEC) appear to encode vectors to nearby objects (Høydal et al., 2019). It is possible to create a spiking neural network that uses raw sensory data to recognize objects and estimate their displacement from the observer, though it remains an active area of research. For example, Osswald et al. (2017) presented a spiking neural network model and neuromorphic demonstration of stereo-correspondence in 3D space. Spiking neural algorithms for object detection (Kim et al., 2020b) and place recognition (Hussaini et al., 2022) have also been developed. Moreover, deep learning has proven to be highly successful in computer vision tasks such as semantic segmentation (Lateef and Ruichek, 2019), and these pre-trained artificial neural networks can be converted to spiking neural networks (Cao et al., 2015). However, in this work, visual processing of raw sensory data is out of scope. Instead, we assume that information regarding distance to landmarks and landmark identity is provided directly as input to SSP-SLAM.

Specifically, we let {*B*_1_, *B*_2_, … } be a set of semantic pointers representing features or landmarks in an environment, at locations {x_1_, x_2_, … }. The input to SSP-SLAM uses these representations to determine the SSP representation of the vector from the agent to each landmark within the agent's field of view, ϕ(x_*i*_−x(*t*)). In short, the input is represented in a population that encodes an egocentric representation of landmark locations that will change over time as the agent passes by landmarks. The neurons in this population have activity patterns like those of object vector cells (OVCs) in the MEC, so we call the population the OVC population. The output of the path integrator and OVC populations are bound together to compute allocentric features locations, $\hat{\phi }\left(\text{x}\left(t\right)\right)⊛\phi \left({\text{x}}_{i}-\text{x}\left(t\right)\right)=\hat{\phi }\left({\text{x}}_{i}\right)\approx \phi \left({\text{x}}_{i}\right)$. This is stored in the object location (OL) population.

**Figure 4:**
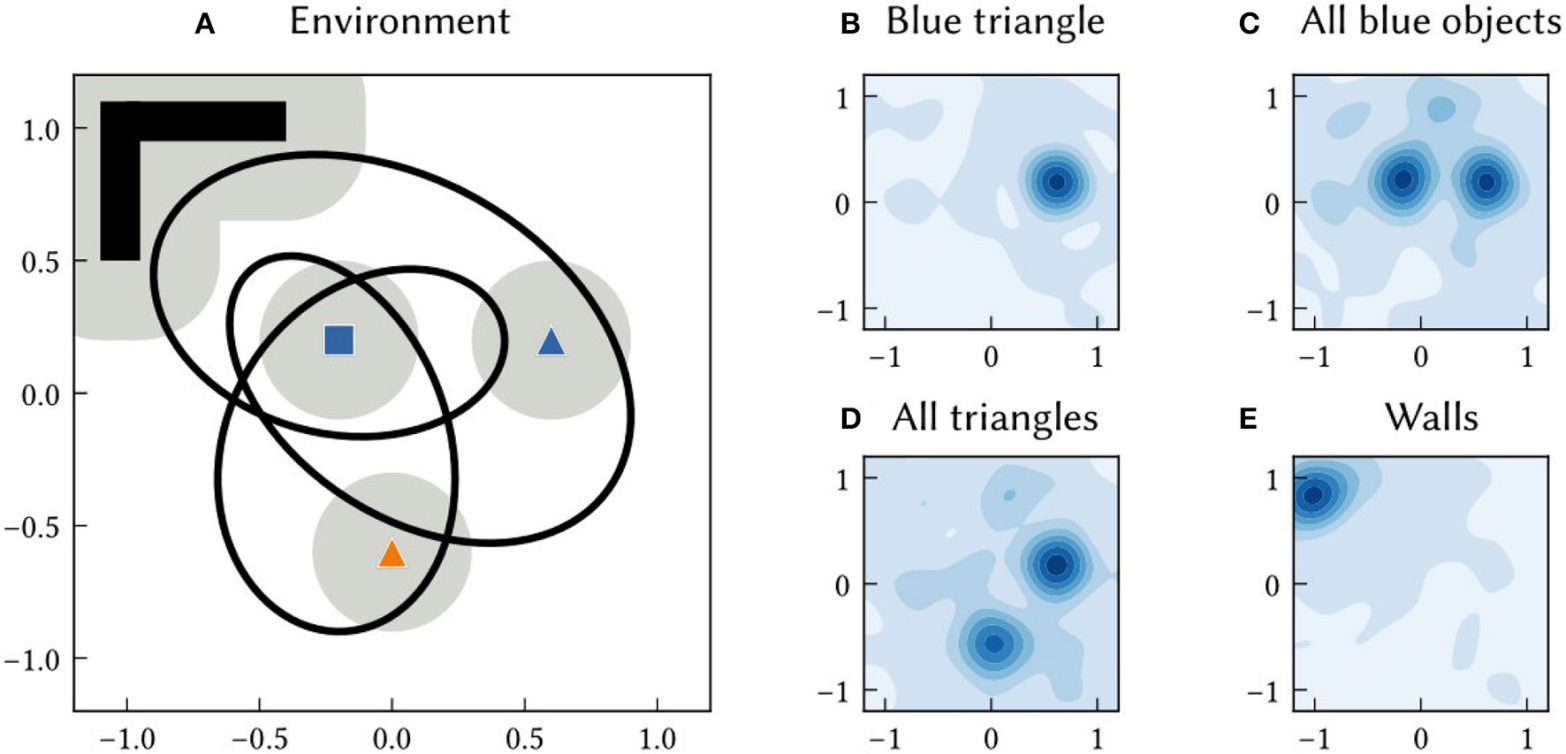
**(A)** An example 2D environment consisting of three point landmarks (a blue square, blue triangle, and orange triangle) and a wall region. An agent transverses a 60 s long trajectory through the environment (the black line). The agent has a limited field of view. The grey circles around landmarks indicates the distance at which the landmark is visible to the agent. SSP-SLAM is used to estimate the agent's location and learn a map of this environment. **(B–E)** The environment map network, trained to map features to locations, is probed at the end of the simulation. In **(B)**, the location of the blue triangle is recalled by passing in *B*⊛*T* to the environment map network. The heat map shows the similarity of the output of the network to SSPs representing points over the 2D. This represents the probability over locations. **(C)** The recall of the location of all blue landmarks, plotted as a similarity map. **(D)** The recall of the location of all triangle landmarks, plotted as a similarity map. **(E)** The recall of the wall area.

As with path integration positions, the allocentric SSP estimate of an landmark location, $\hat{\phi }\left({\text{x}}_{i}\right)$, can be converted into probabilities. The probability density of landmark *B*_*i*_ being at a location x is ${\left(\phi \left(\text{x}\right)\cdot \hat{\phi }\left({\text{x}}_{i}\right)-\xi \right)}^{+}$ (see Figure 4 for examples).

#### 2.2.3. Environment map module

In SSP-SLAM, an environment map is stored in the weights of a heteroassociative memory network. This memory network architecture was first presented in Voelker et al. (2014). It is a neural population that maps input to some desired association. The PES learning rule, given in Equation (13), is used to train the decoders (i.e., the outgoing synaptic weights) of the population. Concurrently, the Voja learning rule, given in Equation (15), is used to modify the population's encoders. This shifts neurons' encoders to be more similar to input they receive. It results in sparser representations in the population, which helps prevent catastrophic forgetting or interference.

Networks that map between landmarks and locations can be thought of as encoding a cognitive map. In SSP-SLAM, several landmark-location mappings are of interest. The associative memory network just described maps features in the agent's field of view (e.g., objects, landmarks, barriers, colors, etc.) to the current estimate of those feature's locations as SSPs, $\hat{\phi }\left({\text{x}}_{i}\right)$. Notably, these environmental features can be structured representations. For example, vector representations of a color, smell, and shape can be bound or bundled together to create a multi-sensory landmark. Using such representations, complex semantic environment maps can be learned.

Other mappings can be used as well. For example, a network can be trained to map feature locations $\hat{\phi }\left({\text{x}}_{i}\right)$, to feature symbols. Or, alternatively, a mapping from feature locations to feature symbols bound with their location, $\hat{\phi }\left({\text{x}}_{i}\right)⊛B$, can be learned. Given an SSP input that represents the whole area of an environment, the network will approximately recall $\underset{i}{\sum }\phi \left({\text{x}}_{i}\right)⊛B_{i}$, and so a single vector representation of a complete map can be recovered. We demonstrate a variety of these mappings in the Section 3.

#### 2.2.4. Loop closure module

The combination of the PI model (presented in Section 2.2.1) and the associative memory network (for environment mapping) provides the core components of a SLAM model. As landmarks are discovered, their perception drives the training of a memory network, which learns a mapping from a symbol-like representation of features, *B*_*j*_, to their locations, $\hat{\phi }\left({\text{x}}_{i}\right)$. When landmarks are re-encountered, the past estimate of their location is recalled by the memory network. This might be different than the current estimate of their locations computed in the OL population, due to errors accumulating in the PI computation. The difference in estimations is used to correct the PI model. This full loop is shown in Figure 2.

## 3. Results

### 3.1. Mapping in 2D environments

In this section, we focus on a single example environment to demonstrate map querying and accuracy in SSP-SLAM. As shown in Figure 4A, we use a simple 2D environment that contains three point landmarks (a blue square, blue triangle, and orange triangle) as well as a wall region. To provide a path, we generate a random, frequency-limited trajectory through the environment and use finite differences to obtain velocities along the path (see Figure 4). The velocity input signal is represented by a spiking neural population, introducing noise to the signal. Model parameters used in this and subsequent experiments (unless stated otherwise) are given Table 1.

**Table 1:** The hyperparameters used for experiments with SSP-SLAM, exceptions are noted in the text.

**Parameter**	**Default value**
Number of neurons	
PI	45,000
GC	1,000
OVC	1,000
OL	27,000
AM	1,000
ME	27,000
Dim of SSPs, *d*	181
View radius of agent	0.3 × env. radius
Post-synaptic time constant, τ_*syn*_	0.05
Max firing rate of LIF neurons	200–400 Hz
Proportion of active neurons	0.1
Voja learning rate	5 × 10^−3^
PES learning rate	1 × 10^−2^

The environment map network is trained to map semantic pointers representing environment features to the feature locations as SSPs. Given the map in Figure 4, it ideally learns the following associations:


(21) $$\begin{array}{ll} \text{BLUE}⊛\text{SQUARE} & \to \phi \left(\left[0.6,0.2\right]\right) \end{array}$$

(22) $$\begin{array}{ll} \text{BLUE}⊛\text{TRIANGLE} & \to \phi \left(\left[0.0,-0.6\right]\right) \end{array}$$

(23) $$\begin{array}{ll} \text{ORANGE}⊛\text{TRIANGLE} & \to \phi \left(\left[-0.2,0.2\right]\right) \end{array}$$

(24) $$\begin{array}{l} \begin{array}{ll} WALL & \to \int_{0.5}^{1.1}\int_{-1.1}^{-0.95}\phi (x,\;y)dxdy \end{array}\\ \;\begin{array}{l} +\;\int_{0.95}^{1.1}\int_{-1}^{-0.4}\phi (x,\;y)dxdy \end{array} \end{array}$$

where BLUE is a semantic pointer representing the color “blue”, SQUARE is the semantic pointer representing the shape “square”, etc.

At the end of the simulation, the actual mapping learned by the environment map network is probed. The locations of particular point landmarks is recalled by feeding in semantic pointer input, e.g., BLUE⊛TRIANGLE as shown in Figure 4B. Additionally, the map was queried for locations of all landmarks sharing certain characteristics. For example, the locations of all blue landmarks was queried by giving the network input BLUE⊛(SQUARE+TRIANGLE) (see Figure 4C).

**Figure 5:**
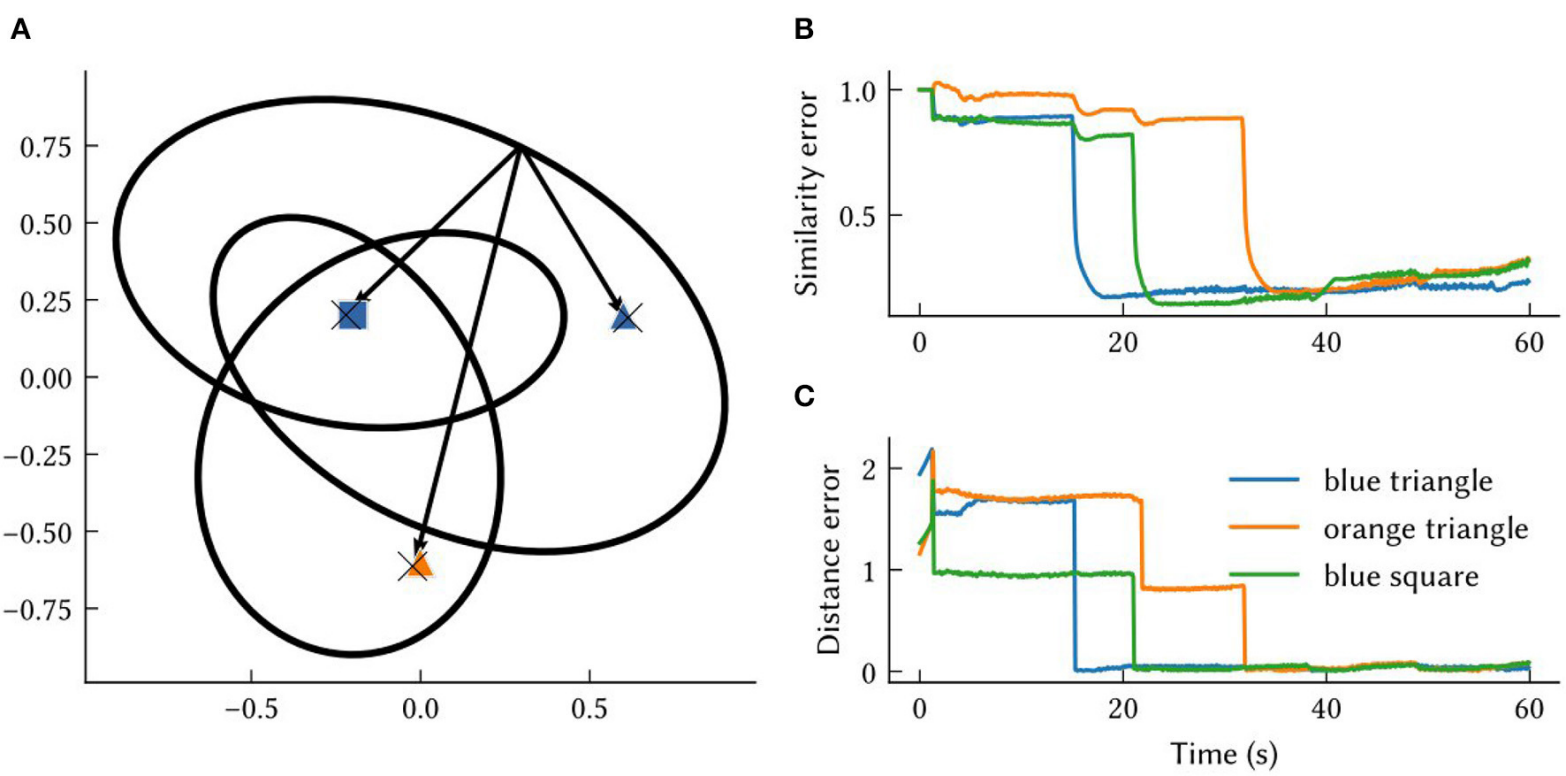
The results from querying vectors to landmarks in the same environment from Figures 4, 6. **(A)** Each “X” marks the model's MAP estimate of a point landmark's location at the end of the simulation. The arrows are estimates of the vectors between self-position and recalled landmarks at the end of the simulation. These approximate vectors are estimated from $\hat{\phi }\left({\text{x}}_{i}-\text{x}\left(t\right)\right)$, obtained by binding the model's other SSP estimates, $\phi {\left(\hat{\text{x}}\left(t\right)\right)}^{-1}⊛\hat{\phi }\left({\text{x}}_{i}\right)\approx \phi \left({\text{x}}_{i}-\text{x}\left(t\right)\right)$. **(B)** The similarity error, $1-\phi \left({\text{x}}_{i}-\text{x}\left(t\right)\right)\cdot \hat{\phi }\left({\text{x}}_{i}-\text{x}\left(t\right)\right)$, over the simulation time *t*. **(C)** The distance between the MAP estimate obtained from $\hat{\phi }\left({\text{x}}_{i}-\text{x}\left(t\right)\right)$ and the ground truth vector between self-position at time *t* and landmark locations, x_*i*_−x(*t*).

In Figure 5A, the MAP estimates of point landmark locations at the end of the simulation are shown. Also plotted is the output of a biologically plausible computation of the vector from the model's self-position estimate to all recalled landmark locations. The output from querying the environment map network for each landmark's SSP location, $\hat{\phi }\left({\text{x}}_{i}\right)$, is combined with the output of the localization module to compute these vectors over the simulation run time. This is done by taking the inverse of the SSP output of the localization module, $\phi {\left(\hat{\text{x}}\left(t\right)\right)}^{-1}$, and binding it with recalled locations from the associative memory, $\phi {\left(\hat{\text{x}}\left(t\right)\right)}^{-1}⊛\hat{\phi }\left({\text{x}}_{i}\right)=\hat{\phi }\left({\text{x}}_{i}-\text{x}\left(t\right)\right)\approx \phi \left({\text{x}}_{i}-\text{x}\left(t\right)\right)$. This produces an estimate of the vector distance between the agent and landmark *i* – a useful quantity for navigation. The error in this computation is plotted in Figures 5B, C. At the beginning of the simulation, environment map has not yet been learned and so the output $\hat{\phi }\left({\text{x}}_{i}-\text{x}\left(t\right)\right)$ is inaccurate. After an item has been encountered, the error drops.

**Figure 6:**
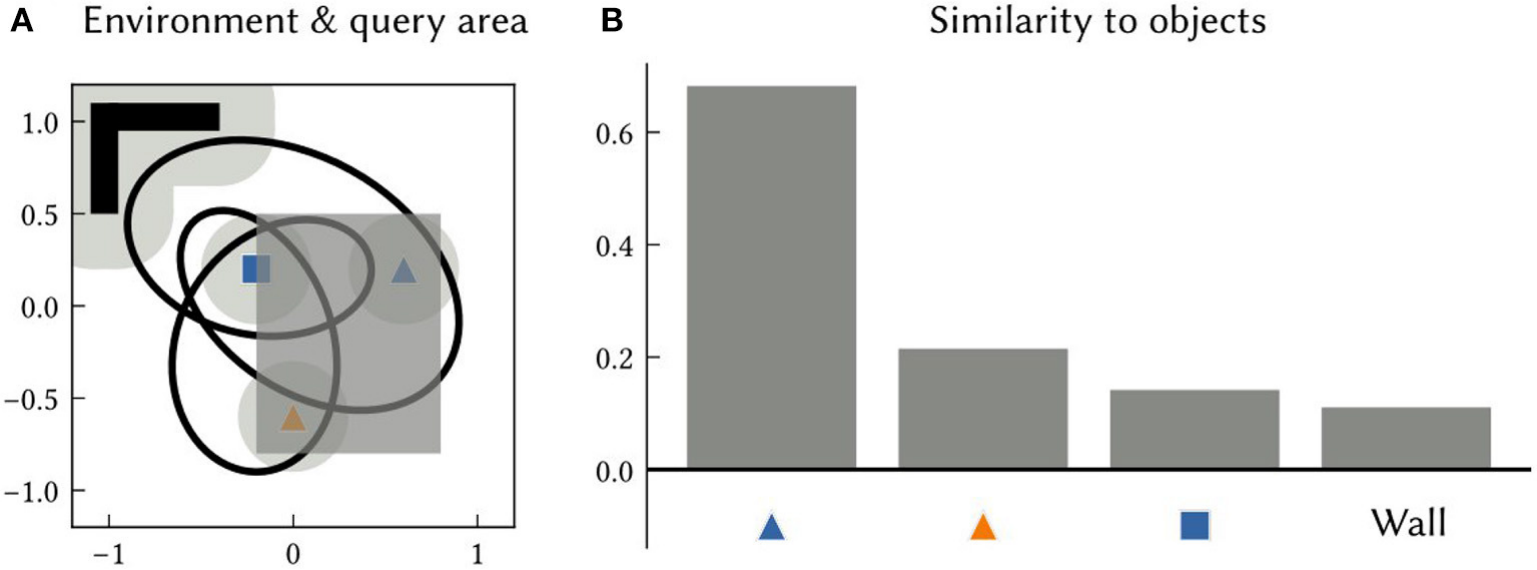
**(A)** An example 2D environment and a query area (the dark grey shaded region). The SSP representing the query area is given as input to an associative memory network that learned to map object location SSPs to object features, using the output of SSP-SLAM's path integrator and OB network components. **(B)** The similarity of the output of the associative memory network to all object semantic pointers in the environment. The results indicate that the orange triangle and blue square are within the queried area.

An associative memory that maps landmark location SSPs to landmark semantic pointers is also trained in this experiment. After learning, SSPs are passed into this network to recall the semantic pointers of landmarks or features at particular locations or over particular areas. An example of querying an area is shown in Figure 6.

### 3.2. Maintaining neural activity patterns

**Figure 7:**
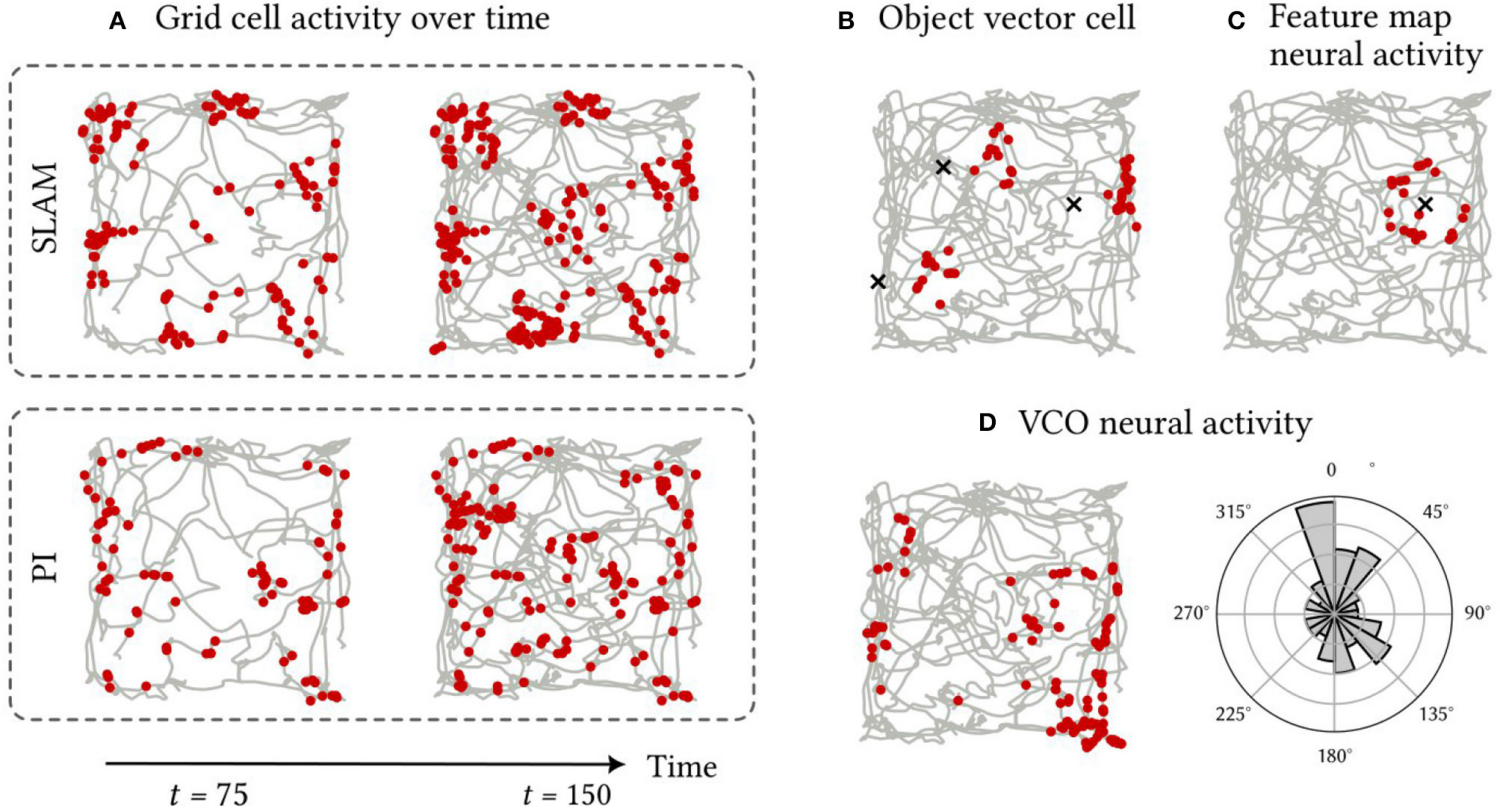
Firing patterns of neurons in SSP-SLAM. The path used for the simulation is shown in grey [obtained from Sargolini et al. (2006)]. Red dots indicate the positions at which a neuron fired. **(A)** A neuron from the GC population encoding $\phi \left(\hat{\text{x}}\left(t\right)\right)$. Spikes are recorded with the normal functioning of SSP-SLAM in the top row. For the second row, error correction from the learned environment map is turned off (i.e., the GC population was representing the output of path integration alone). The first column of both rows presents result half way through the simulation, while the second column displays the complete recording. The corrections from the environment map help maintain the grid-cell-like activity pattern over time. **(B)** A neuron from the OVC population encoding the SSP representation of the vector between x(*t*) and any landmarks in view. Object locations are marked with an “x”. For this simulation, only three landmarks are included for clarity of the visualization. This neuron fires when an landmark is east of the agent. **(C)** A neuron from the associative memory population. The Voja learning rule shifts the neuron's encoder toward its input, resulting in the neuron firing when a particular landmark (marked with an “x”) is in view. **(D)** A neuron from a VCO population in the path integrator. Neurons in this population have conjunctive sensitivity to velocity and position. On the right panel, spike counts are binned by the heading direction of the path, demonstrating the neuron's preference for a particular head direction.

The activity patterns of spiking neurons in various components of the SSP-SLAM are presented and discussed here. SSP-SLAM is run on a 150 s path, recorded from a rat by Sargolini et al. (2006), with ten landmarks at random locations added to the environment for our experiment. Spike trains are recorded from neurons in the path integrator network, GC population, OVC population, and the associative memory network during the simulation. Activity patterns from certain example neurons are shown in Figure 7.

In Figure 7A, we see that a neuron in the GC population indeed has hexagonally patterned activity, as expected. However, this pattern deteriorates when using the path integrator alone. The corrections computed using the trained environment map module ensure the pattern's stability. This environment map is learned by modifying the outgoing connection weights in the associative memory population using the PES rule, while the Voja learning rule is used to modify the encoders of the associative memory population. This results in neurons developing selective sensitivity to particular encountered landmarks, similar to hippocampal place cells (Geiller et al., 2017; Kim et al., 2020a). This is apparent in Figure 7C. In Figure 7B, the activity of a neuron from the OVC population is shown and, as expected, its activity is like that of the object-vector cells of the MEC.

Activity from an example neuron from a VCO population in the path integrator is shown in Figure 7D. Here the spatial sensitivity of the neuron is not discernible. There are no obvious stripe or band patterns, due to the neuron's conjunctive sensitivity to velocity. In a non-random path with correlation between path velocity and position, a stripe pattern would be more apparent (for example, the spiral path example used in Dumont et al., 2022). However, the histogram in Figure 7D showing the distribution of spike counts by heading direction shows that the neuron has selective sensitivity to heading directions between 337.5° and 360° from north. Thus, this neuron is not unlike the head direction cells with conjunctive sensitivity to velocity and position found in the MEC in Sargolini et al. (2006).

### 3.3. Localization in 2D environments

In this experiment, the accuracy of localization in SSP-SLAM is explored. SSP-SLAM is tested on ten different environments. In each environment, ten random locations were chosen for point landmarks, and a two minute-long path generated. The paths are randomly generated from band-limited white noise signals. The model is initialized with the SSP representation of the starting point of the path, and receives the velocity along the path (computed using finite differences) as input over the simulation run time.

**Figure 8:**
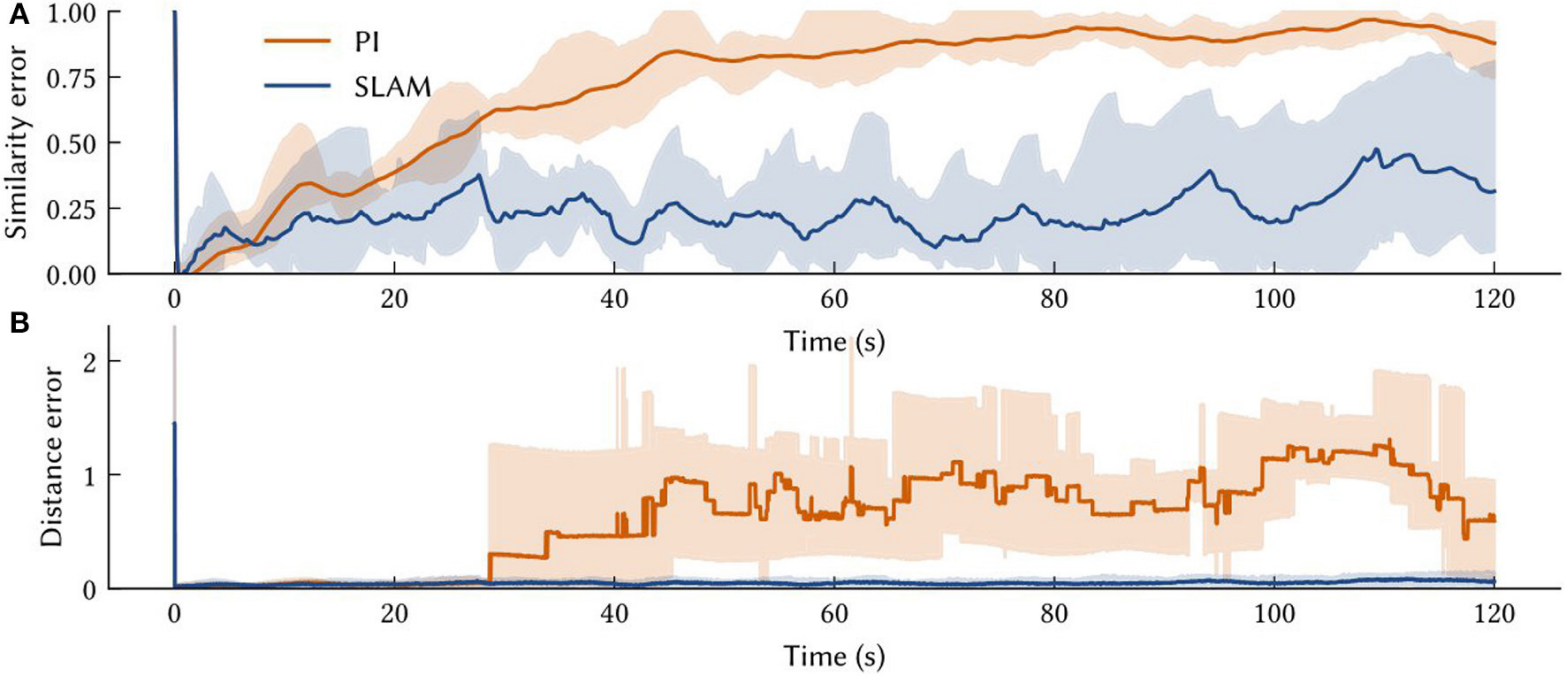
The solid line is the performance measure averaged over ten trials of different paths. Also shown are shaded error bars. **(A)** The similarity error, $1-\phi \left(\text{x}\left(t\right)\right)\cdot \hat{\phi }\left(\text{x}\left(t\right)\right)$, over the simulation time *t* – i.e., how far the off the vector output of the path integrator is from the SSP encoding of the ground truth. **(B)** The distance between the model's MAP estimate of self-position and the ground truth over the simulation time.

To determine the accuracy of the model, the raw spiking data is interpreted as a position estimate as follows (see Figure 8). The vector represented by the path integrator network, $\hat{\phi }\left(\text{x}\left(t\right)\right)$, is decoded from neural activities. Then the $\hat{\text{x}}$ that maximizes ${\left(\phi \left(\hat{\text{x}}\right)\cdot \hat{\phi }\left(\text{x}\left(t\right)\right)-\xi \right)}^{+}$ is computed. This is the MAP estimate of self-position.

The average accuracy of SSP-SLAM localization output is shown in Figure 8. Plotted are similarity and distance errors. The increasing similarity error for SSP-SLAM shows that it is not perfectly representing the SSP encoding of the ground truth. However, the low distance error indicates that an accurate position estimate can be decoded from the output of SSP-SLAM. The absolute trajectory error (the average deviation from ground truth trajectory per time-step) for SSP-SLAM is 0.0529 ± 0.0315 in these experiments. For the PI model alone, this error is 0.7876 ± 0.2958 Integrating the RMSE between SSP-SLAM's MAP estimate and the ground truth over the entire simulation time yields 5.758 ± 3.704 for SSP-SLAM and 73.728 ± 33.69 for PI. The error corrections provided by the environment map in SSP-SLAM result in a more than ten-fold improvement in localization error.

**Figure 9:**
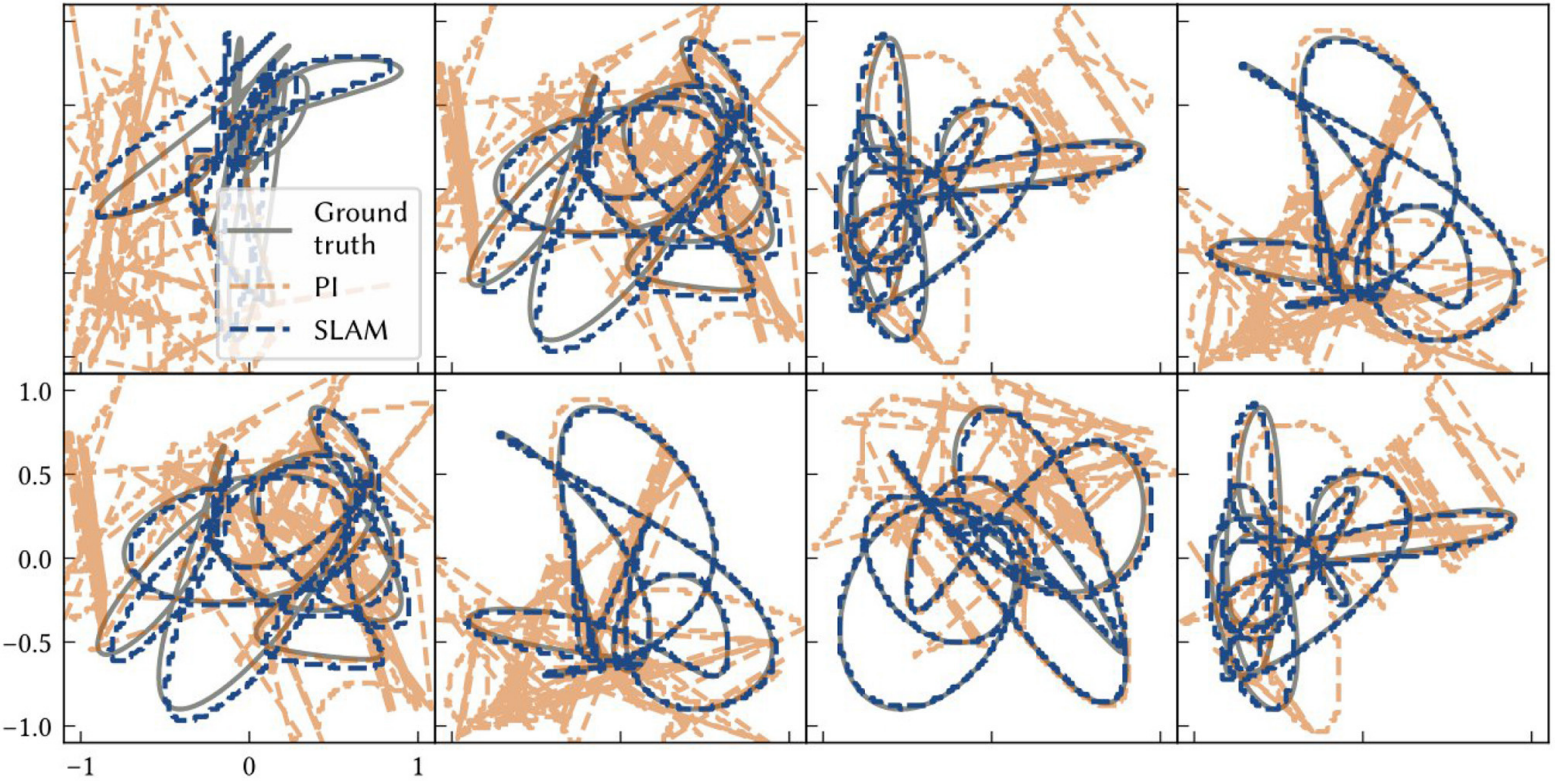
Each panel shows model results for a different environment/ trial. The ground truth paths are plotted as grey solid lines. The dashed blue line is the location estimate from SSP-SLAM. The dashed orange line is the estimate from the path integration network without any corrections from the environment map network (i.e., dead reckoning).

Figure 9 shows examples of the path estimate of SSP-SLAM compared to the exact path and the path integrator network alone (i.e., dead reckoning); the full SSP-SLAM model accurately follows the true path for the entire trajectory. In contrast, the results from the path integrator alone are very poor in these experiments due to the length of the paths and the number of neurons used. Early on in the simulation, the vector represented by the path integrator leaves the manifold in ℝ^*d*^ of the SSPs. Since it is no longer representing a valid SSP, an accurate position cannot be decoded and so the position estimate jumps wildly in the space. In contrast, the corrections computed using the environment map in SSP-SLAM keep the path integrator output near the ideal result.

### 3.4. Localization in 3D environments

**Figure 10:**
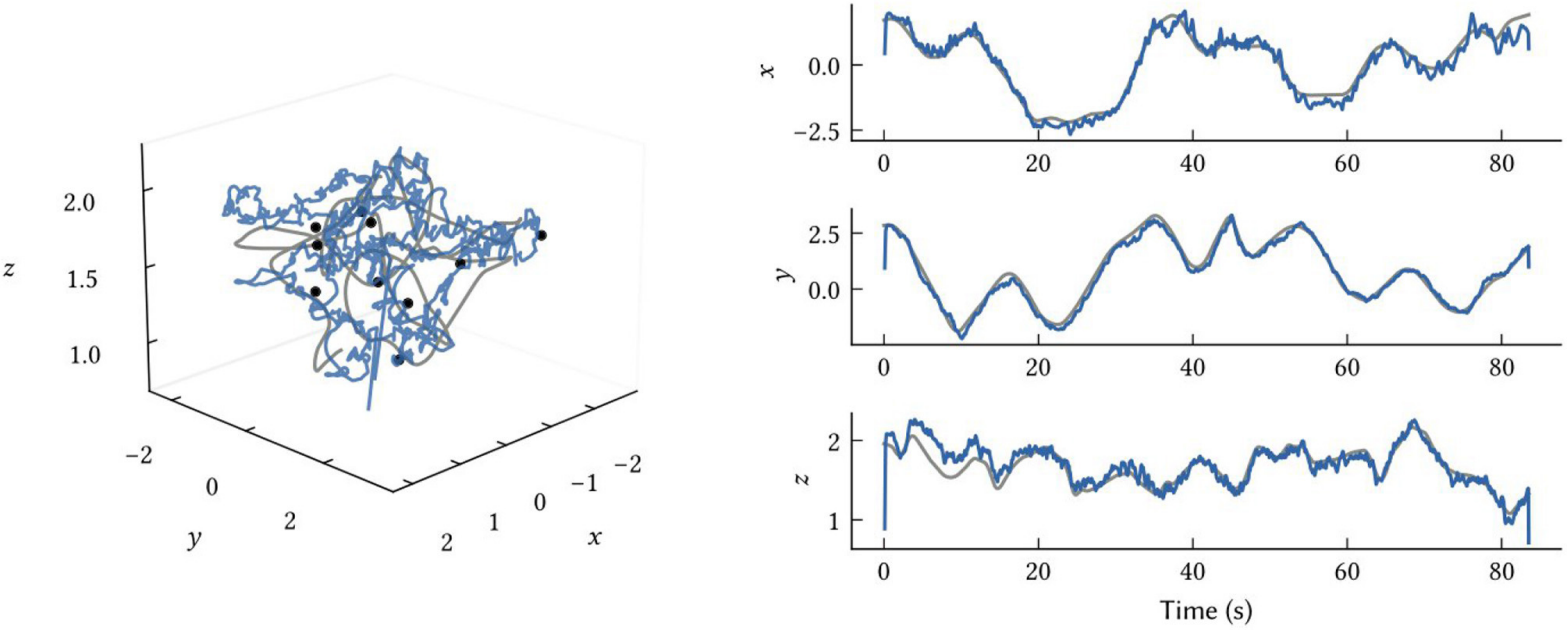
Results from 3D SLAM. The ground truth path is shown in grey, the SSP-SLAM MAP estimate is in blue, and the locations of ten point landmarks are given by the black dots.

While we have focused on 2D environments in this work, the model and all representations naturally generalize to any number of dimensions. In Figure 10, we show how the same model structure using 3D SSPs can be used to accurately perform 3D localization. There are no differences between this and the 2D models, other than using SSP vectors, ϕ(x), encoding x∈ℝ^3^.

### 3.5. Neuromorphic simulation of dead reckoning

**Figure 11:**
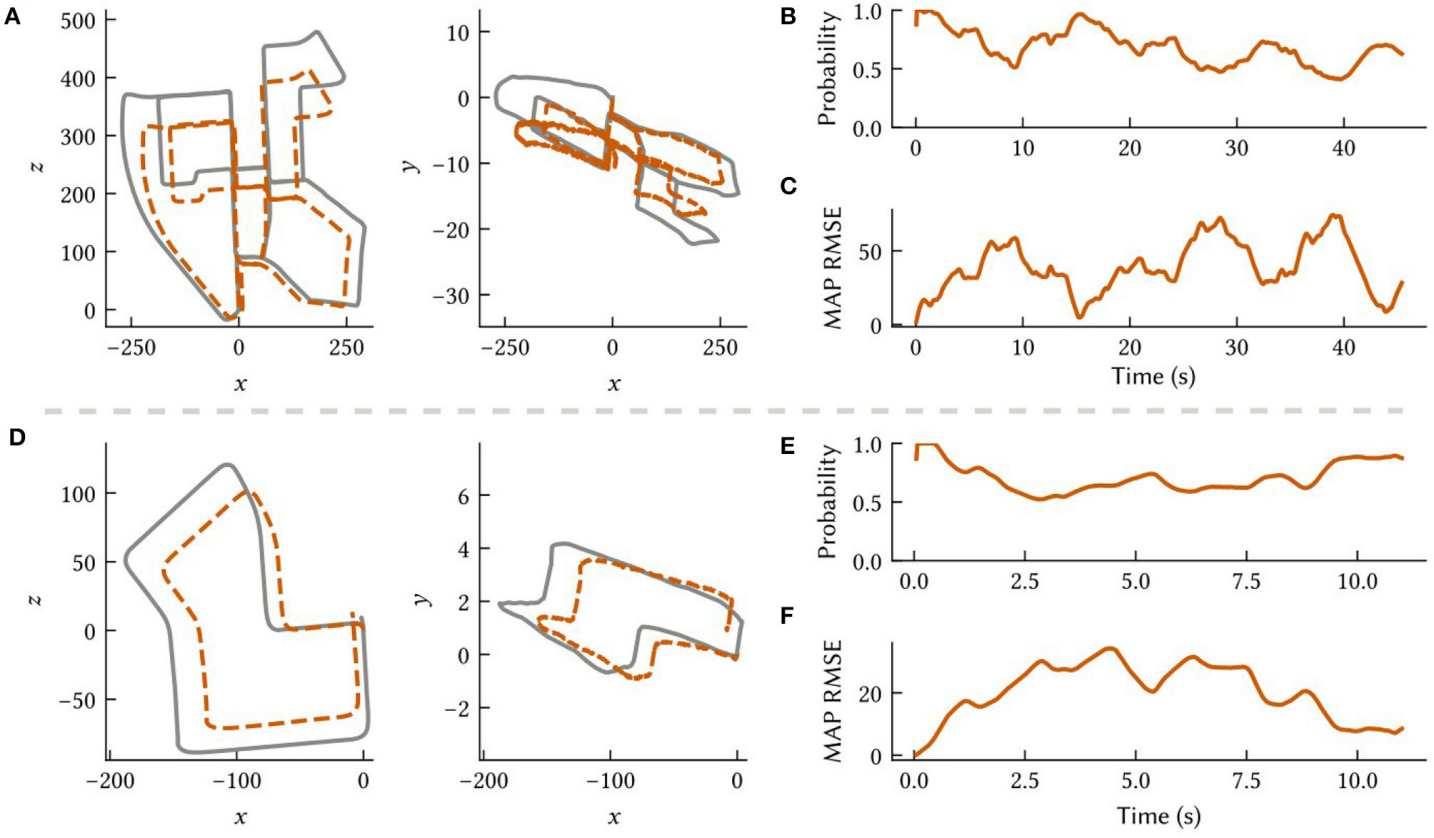
Results from the path integrator network simulated on the NengoLoihi emulator using two different 3D paths constructed from the KITTI odometry dataset (Geiger et al., 2012). **(A, D)** Comparison of the ground truth paths (in grey) to our model's MAP estimate (in orange) along different dimensions. **(B, E)** The probability the model assigns to the ground truth over time. **(C, F)** The RMSE between the MAP estimate and ground truth.

To investigate the feasibility of deploying SSP-SLAM on neuromorphic hardware, we simulated the path integrator network on the NengoLoihi emulator. This Python package allows spiking neural network models built in Nengo to be run on Intel's Loihi architecture. It includes both support for running models on the Loihi hardware and a Loihi emulator, which we used for these experiments. In this experiment, we run the model on paths derived from the KITTI odometry benchmark (Geiger et al., 2012). However, we do not use raw visual input from the KITTI datasets, as our model does not support visual SLAM. Rather, we use velocity signals computed via finite differences on the ground truth paths and represented by a neural population. To compensate for the absence of the landmark perception, environment map, and loop closure modules, the total number of neurons in the path integrator was increased to 90,000 to reduce drift. Results are shown in Figure 11.

Notably, the NengoLoihi emulator implements the same limited precision mathematics as the actual hardware, using 8-bit weights and a quantized neuron response function. Figure 11 shows that the path integrator network is robust to these additional constraints, and continues to perform largely as expected, although with more error compared to the typical performance of the full SSP-SLAM model (Section 3.3).

## 4. Discussion

### 4.1. Prior research

The development and implementation of SLAM algorithms for mobile robots has garnered significant attention in academic and engineering communities. Approaches generally involve recursive Bayesian estimation—via various kinds of Kalman Filters (Smith et al., 1990; Brossard et al., 2018), Particle Filters (Montemerlo et al., 2002; Sim et al., 2005), or occupancy grid methods (Stachniss et al., 2004)—or graph optimization (Thrun and Montemerlo, 2006; Sünderhauf and Protzel, 2012). In recent years, researchers have focused on incorporating semantic information into SLAM systems, using deep artificial neural networks, particularly convolutional or recurrent neural networks for object detection and semantic segmentation. The use of semantic information in SLAM has been found to improve performance and robustness of robot localization (Frost et al., 2016; Stenborg et al., 2018; Bowman, 2022). Furthermore, robots equipped with semantic SLAM hold the promise of performing higher-level tasks, such as planning paths based on human instructions that reference objects in the environment. Concurrently, an alternative approach to SLAM, drawing inspiration from the brain, has continued to develop novel algorithms with the goal of improving efficiency and robustness (Milford et al., 2004, 2016; Silveira et al., 2015; Yu et al., 2019). In this line of research, models of neural path integration inspired by hippocampal cells are used for localization. Coupling such neural algorithms with recent developments in neuromorphic hardware, as we have done here, aims to both improve our understanding of how the brain accomplishes SLAM and to improve the power efficiency of engineered solutions.

Neural localization models used in this alternative approach can generally be divided into two categories: Continuous Attractor Network (CAN) models (Samsonovich and McNaughton, 1997; Tsodyks, 1999; Conklin and Eliasmith, 2005) and Oscillator-Interference (OI) models (O'Keefe and Burgess, 2005; Burgess et al., 2007; Hasselmo et al., 2007; Welday et al., 2011). In CAN models, path integration is performed by a recurrently connected neural sheet, whose dynamics sustain a single Gaussian-like activity bump that represents the self-position estimate of an agent. In contrast, in OI models, the self-position estimate is encoded by the phase differences between Velocity-Controlled Oscillators (VCOs)—oscillators whose frequency is modulated by a velocity signal.

The seminal application of neural-inspired methods to SLAM is RatSLAM, in which visual odometry is used to drive a CAN (Milford et al., 2004, 2016). The CAN consists of “pose cells” (similar to the place and head direction cells found in the hippocampal formation) and maintains an estimate of self-position and orientation. Sensor data is processed outside the neural network to create a template array (for example, raw visual input is converted to an intensity profile vector). When a novel template is observed, a new “local view cell” (similar to the spatial view cells in the hippocampus) is added to the network. The population of these cells is sparsely connected to the CAN, with associations learned via Hebbian learning. Additionally, a graph is constructed and updated with a graph relaxation algorithm online to create a topological environment map. Its nodes store experiences in the form of activity of pose cells and local view cells along with robot pose estimates.

In contrast, a hybrid OI-CAN model is used for path integration in SSP-SLAM and a graphical environment map is not learned—instead, the outgoing connection weights from the memory network implicitly store a map which can be retrieved by querying the network. The Voja rule, which is used to shift the associative memory population encoders toward observed input in SSP-SLAM, plays a similar role to the template novelty detection and addition of local view cells that occurs in RatSLAM. Furthermore, we have not implemented an external module for pre-processing of sensory data, and we use landmark semantic pointers and displacement SSPs in lieu of templates. Object detection and depth estimation algorithms would be required to obtain this input from visual data.

Many models have since extended the original RatSLAM. CAN SLAM models with place cell-like activity were also used by BatSLAM (Steckel and Peremans, 2013), an extension to RatSLAM for handling environment information from sonar sensors, and DolphinSLAM (Silveira et al., 2015), developed for 3D SLAM in underwater environments. A CAN consisting of conjunctive grid cells was used in the SLAM model presented in Zeng and Si (2017). Three-dimensional SLAM in realistic environments with grid cells was also explored in NeuroSLAM (Yu et al., 2019). Unlike our work, none of these models use spiking neural networks.

More recent research has focused on developing spiking networks for SLAM and testing them on neuromorphic hardware. Spiking 2D SLAM models were presented in Tang and Michmizos (2018), Tang et al. (2019), and Kreiser et al. (2020a,b). In Kreiser et al. (2020a), a SLAM system on the Loihi chip was used to estimate the head position of an iCub robot as it visually explored a wall with a dot pattern acting as the environment. Tang et al. (2019) made use of a depth camera and Bayesian updates on a posterior distribution represented by neural population. They found that their SLAM system, when run on Loihi, was more energy efficient by two orders of magnitude compared to a baseline method on a CPU. While the models discussed here use raw sensory input, it should be noted that non-spiking visual modules are used to process this information and obtain input for SLAM. For instance, intensity profile vectors or feature colors and distances from the observer are used. In contrast to SSP-SLAM, none of the models mentioned incorporate any elements of OI to perform path integration, or perform 3D SLAM. Furthermore, some of these models employ “localist”/discrete representations, such as using one neuron to represent each integer value for heading direction or discretized distance to features. This approach does not support generalization and does not scale well to higher dimensional representations, unlike SSPs.

Taken together, and summarized in Table 2, past work provides examples of spiking and non-spiking networks, using CANs for path integration. However, unlike SSP-SLAM, none of these approaches provides a methodology for incorporating semantic information or for online learning of semantic environmental maps. In addition, none of these employ SSPs, or the same combination of a OI-CAN network in a fully spiking model capable of functioning equally well in both 2D and 3D spatial environments, as demonstrated above.

**Table 2:** Comparison of bio-inspired SLAM models.

**Model**	**Sensors**	**Input representation**	**Dim**.	**Localization**	**Env. map**	**Cells**	**Experiment scale**	**Spiking**	**Neuromorhpic hardware**
SSP-SLAM	None	Displacement to features as an SSP and feature identities as SPs	Any, tested on 2D & 3D	OI-CAN hybrid	Weights between landmark population to landmark locations	HDC, GC, landmark cells, OVC	Small	Yes	Partially
RatSLAM (Milford et al., 2004, 2016)	Monocular camera	Greyscale image intensity profile	2D	CAN	Topological map associating local views with position stored as a graph	Pose cells, local view cells	Large	No	No
BatSLAM (Steckel and Peremans, 2013)	Biomimetic sonar	Intensity difference between left and right Echolocation Related Transfer Functions	2D	CAN	Topological map local views with position stored as a graph	Pose cells, local view cells	Small	No	No
DolphinSLAM (Silveira et al., 2015)	Sonar & visual	One-hot representation obtained from FabMAP algorithm on top of a Bag of Words model	3D	CAN	Graph with nodes storing local view, place cell and position while edges store displacements	3D PC, local view cells	Small	No	No
NeuroSLAM (Yu et al., 2019)	Panoramic camera	Greyscale image intensity profile	3D	CAN	Topological map storing activities of local view cells, GCs, HDCs, and estimated pose	3D PC, conjuctive 3D GC and HDC, local view cells	Large	No	No
Kreiser et al. (2020a)	Event-based camera	Detection of blinking LEDs at different frequencies	2D	CAN	Weights from landmark population to a HDC population	HDC, landmark cells	Small	Yes	Fully
Tang et al. (2019)	RGB-Depth camera	Discretized distances to landmarks	2D	CAN	Weights from PC to a displacement-from-border population	2D PC, HDC, border cells, Bayesian cells	Small	Yes	Fully

### 4.2. Performance

We have presented the results of several experiments on SSP-SLAM to assess its performance and utility. The model demonstrates accurate localization capabilities on different paths, both two-dimensional and three-dimensional. To achieve this, a hybrid OI-CAN model is employed for path integration. Notably, this is the only SLAM model (to our knowledge) that uses OI techniques for localization. This approach has the advantage of easy generalization to higher dimensional spaces. Typically, CAN models describe a neural population as a 2D sheet or 3D array (often with periodic boundary conditions), where the geometry specifies the recurrent connectivity pattern required for localization. However, this only supports unimodal position estimates, and the connectivity pattern must be modified and made more complicated to move to higher dimensional path integration.^1^ In contrast, in our approach the recurrent connectivity of the path integrator network remains the same regardless of spatial dimensionality. This allows the same model to switch seamlessly between SLAM in different spaces and domains.

Furthermore, SSP-SLAM encodes environment maps in the outgoing connections of an associative memory network, which are learned online using biologically plausible learning rules. The map generated is a semi-metric, semantic map that uses symbol-like vector representations that have been leveraged in a variety of large-scale cognitive models (Eliasmith, 2013; Arora et al., 2018; Kajić et al., 2019; Kelly et al., 2020; Gosmann and Eliasmith, 2021). By working in the SSP and VSA paradigm, we are able to formulate the problem in such a way that unites metric and semantic SLAMs. This approach unites analytical models of vehicle motion and map construction with neural networks, resulting in a formulation that is compatible with modern ML approaches to robotics, while still maintaining the explainability of the system. These feature distinguishes SSP-SLAM from other bio-inspired SLAM models and makes it the first spiking semantic SLAM model to our knowledge.

This inclusion of semantic information helps SSP-SLAM be more accurate. Specifically, SSP-SLAM performs loop closure via corrections to the PI network provided by the environment map, which leads to significant improvements in localization accuracy. After training, the map can be queried to obtain object locations given their symbol-like representation as a semantic pointer. Alternatively, item representations can be obtained by querying specific areas, or vectors between the agent and landmarks can be computed. These kinds of direct queries of semantic map knowledge cannot be easily made with past spiking network map representations.

Finally, a key element of the model, the path integrator, was tested on a neuromorphic emulator. The results indicate that the model can maintain expected accuracy (given the absence of error correction mechanisms) on neuromorphic hardware. Notably, all additional operations used in the model have been implemented on neuromorphic hardware in other work (Knight et al., 2016; Mundy, 2017), so we believe this demonstration strongly suggests that a full neuromorphic implementation is achievable. Overall, this study presents a novel and promising approach to SLAM based on a fully spiking neural network.

### 4.3. Limitations

This study presents a novel model that employs biologically-inspired mechanisms to solve SLAM. However, SSP-SLAM has several limitations. First, the full SSP-SLAM model has not been tested on a neuromorphic chip emulator nor has the model been deployed on an actual neuromorphic hardware platform. Second, the model was tested on a small scale and artificial environments, which restricts what conclusions we can draw as to its generalizability to more complex, real-world environments.

To improve the model's utility, it is essential to test it on real-world input and integrate it with a network that can process raw sensory data. Such an approach would enhance the model's ability to handle more complex and diverse environmental conditions. Moreover, the current model's accuracy is inferior to that of non-biologically inspired SLAM methods, which limits its usefulness to mobile robotics. This accuracy drop and the use of small scale test environments is true of current spiking SLAM models more generally. Despite this, the use of neuromorphic computing and hardware has the potential to improve energy efficiency of SLAM systems, which is particularly useful in mobile robotics applications. This encourages further research into spiking SLAM systems. Reduced power demands permits the deployment of SLAMs in progressively more power-constrained environments, such as edge computing or operations in GPS-denied settings, like space or sub-sea exploration. An increasing number of algorithms have harnessed the advantages of spike-based computing to make gains in efficiency and speed (Yakopcic et al., 2020; Davies et al., 2021; Yan et al., 2021).

Therefore, while the current model shows promise in enabling biologically-inspired SLAM, its limitations in terms of testing and accuracy should be addressed before considering its wider application in real-world scenarios. Further research could focus on testing the model on larger networks and more complex environments, as well as investigating ways to improve its accuracy.

### 4.4. Future work

One clear direction for future work is ameliorating the limitations discussed in the previous section. Beyond this, there are several other directions that warrant further exploration—for example, explicit modeling of sensor uncertainties using SSPs, introducing coupling dynamics to increase localization accuracy, higher-dimensional SLAM, and integration with other cognitive models.

Accurate of localization is vital and phase drift is one of the main factors contributing to SSP inaccuracy. As path integration progresses, errors can accumulate in the phases of the velocity-controlled oscillators (VCOs), resulting in inconsistencies that degrade the spatial information (e.g., see Figure 8). The loop-closure error corrections (Figure 2) can shift the phases toward the true values, but the phase inconsistencies would still be present. However, one could take advantage of the redundancy in the SSP representation by adding coupling between the VCOs that enforce their proper phase relationships (Orchard et al., 2013).

Additionally, higher-dimensional SLAM could be a promising area of investigation. The proposed model can be extended to localization and mapping in any dimension of space by modifying the input without changing the model or hyperparameters. Although SLAM is mainly applied to navigation and mapping in physical spaces, operating in dimensions equal to or less than three, it is possible that the same neural mechanisms underlying spatial navigation and mapping could be applicable to non-spatial domains, such as mapping in high-dimensional conceptual space. The idea that similar computations to those behind SLAM may be understood as core cognitive processes has been proposed in Safron et al. (2022).

The application of SSP-SLAM to localization and mapping in various spaces (including non-spatial ones) via interactions with other cognitive systems is promising area for future research. By employing control mechanisms to manipulate the input to SSP-SLAM, it may be possible to model different cognitive functions. For instance, one could switch between motion input from sensory systems to perform localization and input from memory and cognitive maps to simulate path replay or planning. This could be realized by integrating SSP-SLAM with more complex memory, action selection, and reasoning systems. Since the proposed model was developed using the SPA, it fits naturally within the context of NEF and other SPA models, including Spaun (Stewart et al., 2012a; Choo, 2018). Integration of the proposed SLAM model with other models constructed with these tools could be used to develop systems equipped with more sophisticated cognitive capabilities and able to tackle multiple tasks. Exploiting memory and reasoning capabilities in large spatial environments remains a challenge for models of biological cognition.

### 4.5. Summary

In conclusion, we have proposed a novel spiking semantic SLAM model, SSP-SLAM, which is inspired by the hippocampal formation in the mammalian brain. The model is unique in its integration of a hybrid OI-CAN path integrator, online biologically-plausible learning of an environment map, and use of symbol-like object representations in a spiking network. This combination enables the model to perform SLAM accurately in small scale environments and learn representations that can be queried in powerful ways. For example, it can provide information about what is located in a particular area of the map, report vectors between landmarks, and identify the location of objects based on their properties, such as their color. Furthermore, these techniques advance the sophistication of biologically plausible SLAM networks, showing a wide variety of previously identified cell types while demonstrating functionality in 2D and 3D environments.

Finally, we have tested a core component of the network on a neuromorphic hardware emulator, which represents an important step toward achieving a full system running on neuromorphic hardware. While significant work remains to achieve this goal, we believe that the methods and components employed in this study provide a foundation for future research in this area. With continued progress, this spiking semantic SLAM model could have important applications in a wide range of fields, including robotics, artificial intelligence, and neuroscience.

## Data availability statement

The raw data supporting the conclusions of this article can be found here: https://github.com/nsdumont/Semantic-Spiking-Neural-SLAM-2023.

## Author contributions

ND, JO, and CE contributed to the theoretical development and conception of this work. ND wrote the code used in experiments, generated results and figures, and wrote the first draft of the manuscript. PF developed the probabilistic interpretation of SSPs used in this manuscript. All authors contributed to manuscript revision, read, and approved the submitted version.

## Footnotes

^1^Recent research has explored variants to traditional CANs that overcome these limitations. A multimodal CAN model was presented in Wang and Kang (2022) and research exploring CANs with arbitrary dimensional attractor manifolds and more biologically realistic asymmetries in synaptic connectivity was presented in Darshan and Rivkind (2022). Such CAN variants have not been used in SLAM systems.
